# Rural–urban differences in the perceived impact of COVID-19 on mental health by European women

**DOI:** 10.1007/s00737-024-01443-3

**Published:** 2024-02-13

**Authors:** Mercedes Rodríguez, José A. Camacho

**Affiliations:** Institute of Regional Development, University of Granada, Calle Rector López Argüeta. Edificio Centro de Documentación Científica, 3ª Planta, 18071 Granada, Spain

**Keywords:** Women, Mental health, Urban, Rural

## Abstract

**Purpose:**

Many studies have documented an adverse impact of the pandemic on women´s mental health. This cross-sectional study aims to explore associations between women's perceived impact of lockdowns and curfews on their mental health and their residential location, along with other contextual and individual factors.

**Methods:**

Using data from the Flash Eurobarometer 2712 “Women in times of COVID-19”, conducted between January 25 and February 3, 2022, across the 27 Member States of the European Union (n = 23,671), this study applied bivariate tests and stratified models based on respondent location (rural areas, small or medium-sized towns and urban areas). The exploration sought predictors influencing the perceived mental health impact, encompassing five individual characteristics (age, disability, employment status, educational attainment, and household type), perceptions of violence against women, and country of residence. The dependent variable was assessed subjectively, measured on a scale from 1 (minor negative impact) to 5 (major negative impact).

**Results:**

Women living in urban areas generally reported a higher perceived negative impact on mental health compared to women in rural areas or in small/medium-sized towns. Age and disability were significantly linked to perceiving a negative impact on mental health. Similar adjusted odds ratios for age were observed across rural areas (aOR 0.97, 95% CI = 0.97–0.98), small or medium-sized towns (aOR 0.98, 95% CI = 0.97–0.98), and urban areas (aOR 0.97, 95% CI = 0.97–0.98). In terms of disability, the odds were higher in rural areas (aOR 1.44, 95% CI = 1.20–1.73) than in urban ones (aOR 1.36, 95% CI = 1.15–1.62). Among women residing in urban areas, those in childless couples were less likely to perceive a negative impact on mental health (aOR 0.89, 95% CI = 0.80–0.99) compared to women in couples with children. Respondents perceiving increased violence against women due to COVID-19 were more likely to perceive a negative impact on mental health, with higher odds ratios in rural areas (aOR 1.56, 95% CI = 1.40–1.74) compared to urban areas (aOR 1.29, 95% CI = 1.17–1.41). Differences across countries were also found.

**Conclusion:**

The perceived impact of lockdowns and curfews on mental health exhibited variance between urban and rural areas. These disparities were influenced by individual characteristics such as age, disability, or household type, as well as the effects of COVID-19 on violence against women and contextual variables like country of residence.

## Introduction

Understanding the association between residential settings and mental well-being has been a subject of interdisciplinary research in recent years (Curtis 2010). Several studies have investigated mental health disparities between rural and urban areas, often identifying higher mental health challenges in urban settings (Breslau et al. 2014; Kovess-Masféty et al. 2005; Peen et al. 2010; Peterson et al. 2009). Peterson et al. (2009) categorized explanatory factors for these disparities into two main types, individual and contextual. Contextual factors encompass domains such as the structure and availability of health care resources (which help individuals to resolve mental health issues), local economic conditions (such as income or employment levels), social disruption (reflecting the social environment), and social capital (representing trust, reciprocity, or civic engagement), all of which have implications for mental health disparities.

Estimations on COVID-19's impact across EU territories (Kapitsinis 2020; Natale et al. 2023), indicate that while urban areas initially faced a quicker spread and higher mortality, subsequent waves have shown less distinct differences (Natale et al. 2023). Recent studies point to potential differential impacts on mental health between rural and urban areas (Desdiani et al. 2022; Henning-Smith et al. 2023; Jia et al. 2021; Liu et al. 2021; Monnat 2021; Pérès et al. 2021). Initial studies indicated heightened suffering among rural residents, potentially due to limited mental health services or lower socioeconomic status (Jia et al. 2021; Monnat 2021). However, further analyses presented different findings (Desdiani et al. 2022; Henning-Smith et al. 2023), suggesting a potentially lower negative impact of the pandemic on mental health in rural areas, possibly attributed to robust social support systems (Liu et al. 2021; Pérès et al. 2021). For instance, in France, older adults in rural areas reported better experiences during the first lockdown due to enhanced social support and family presence (Pérès et al. 2021). In contrast, urban residents in China reported more mental health issues related to the pandemic than rural residents (Liu et al. 2021).

Examining the impact of COVID-19 restriction measures, it's apparent that these measures might be associated with adverse mental health effects (Adams-Prassl et al. 2022; Borrescio-Higa and Valenzuela 2021; Etheridge and Spantig 2022; Oreffice and Quintana-Domeque 2021; Pieh et al. 2020; Proto and Quintana-Domeque 2021; Simha et al. 2020). Research confirms a gender-differentiated impact of COVID-19 on women, with women experiencing a more pronounced decline in mental well-being compared to men (Connor et al. 2020; Devoto et al. 2022; Riecher-Rössler 2022; Wade et al. 2021). Notably, lockdown orders in the US notably widened the gender gap in mental health by 61% (Adams-Prassl et al. 2022). European studies, such as the Survey of Health, Ageing and Retirement in Europe (SHARE), revealed that women had higher odds of worsened mental health compared to men during the pandemic (Scheel-Hincke et al. 2021; Wester et al. 2022). This was attributed in part to increased care responsibilities and changes in employment status, where women faced higher job loss rates than men (Borrescio-Higa and Valenzuela 2021; Etheridge and Spantig 2022; Oreffice and Quintana-Domeque 2021). Thus, women in employment during the pandemic had higher mental health levels than women who lost or left paid work (Wang et al. 2022). Additionally, women, predominantly the caregiving workforce in healthcare and domestic settings (Llena-Nozal et al. 2022), faced increased vulnerability to infection and transmission during the pandemic (Connor et al. 2020; Wade et al. 2021).

Despite these findings, research explicitly examining the differential impact of the pandemic on women's mental health across the rural–urban continuum is scarce. This study fills this critical gap by exploring the association between women's perceived impact of lockdown and curfew measures on their mental health, considering their residential location alongside contextual and individual characteristics. The study utilizes comprehensive representative data from women residing in the European Union (EU).

## Material and methods

### Data source

Data for this study were obtained from Flash Eurobarometer 2712, titled “Women in times of COVID-19”, conducted by Ipsos European Public Affairs, a multinational market research firm specializing in multi-country studies for intergovernmental and international organizations (GESIS 2022). The survey was conducted between January 25 and February 3, 2022, and encompassed a representative sample of 26,741 women aged 15 years and above from all 27 Member States of the European Union (EU). The sample distribution across countries was as follows: Austria: 1,066; Belgium: 1,122; Bulgaria: 1,023; Cyprus: 531; Czech Republic: 1,041; Germany: 1,088; Denmark: 1,053; Estonia: 1,015; Spain: 1,083; Finland: 1,043; France: 1,083; Greece: 1,058; Croatia: 1,017; Hungary: 1,037; Ireland: 1,029; Italy: 1,153; Lithuania: 1,039; Luxembourg: 520; Latvia: 1,043; Malta: 538; The Netherlands: 1,007; Poland: 1,043; Portugal: 1,001; Romania: 1,026; Sweden: 1,029; Slovenia: 1,014; and Slovakia: 1,039.

The interviews were conducted through Computer-Assisted Web Interviewing (CAWI) using Ipsos online panels to ensure statistically valid representation across the populations of each EU Member State. Panel recruitment involved mail invitations sent to random households in each Member State, eliminating voluntary participation. All surveys were administered online to maintain consistency and minimize mode effects that could influence respondent answers based on the mode of administration.

The comprehensive data collected in Flash Eurobarometer 2712 captures women's opinions on the pandemic's impact on mental health, violence against women, and women's professional lives. Sampling quotas were established based on age groups (15–24, 25–34, 35–44, 45–54, 55–64, and 65 years and older) and geographic regions, considering country size and regional distribution. Following data collection, a post-stratification weighting procedure was employed to align the sample with selected population totals. Random Iterative Method (RIM) weighting was used to adjust for marginal age by gender, activity status, and regional population distributions.

### Measurement

The survey collected responses regarding the perceived negative impact of lockdown and curfew measures on mental health over the past two years. Respondents used a 1 to 5 scale, ranging from “minor negative impact” (1) to “major negative impact” (5) in answer to the question: “Since the beginning of the COVID-19 pandemic, governments have taken various measures to stop the spread of the virus. On a scale from 1 to 5, to what extent did lockdown and curfew measures, limiting your options to shop, go out, go to events, etc. have a negative impact on your mental health?”.

Furthermore, participants indicated their residential locations based on urban/rural distinctions: “Would you say you live in a rural area or village, a small or medium-sized town or a large town/city?”. This classification aligns with the United Nations Statistical Commission's application of the degree of urbanization, categorizing territories along the rural–urban continuum into three distinct classes: rural areas, towns and semi-dense areas, and cities (endorsed by the European Commission et al. (2021).

We considered various individual factors known to influence mental health (Caycho-Rodríguez et al. 2021). These factors included age, disability (measured through general activity limitation, encompassing difficulties in hearing, seeing, walking, etc.), employment status (self-employed, employee, manual worker, not working), educational attainment (categorized as never in full-time education, primary, secondary, or tertiary education), and household type (couple with children, couple without children, single parent with children, single without children, multi-generational household, co-living or other forms of communal living).

In addition to these individual factors, we incorporated a binary variable reflecting respondents' perceptions of the pandemic's impact on violence against women. The survey question asked: “Do you think that the COVID-19 pandemic has led to an increase or decrease in physical and emotional violence against women in your country?”. We coded the variable as 1 for 'increase' and 0 for any other response. Lastly, to account for contextual factors, binary variables corresponding to the participant's country of residence were included in the analysis.

### Statistical analysis

The analysis was conducted using Stata 16 (Stata Corp LP, College Station, TX). The respondents served as our unit of analysis and all analyses were stratified by rural/small-medium/urban location.

To identify differences in individual factors, we conducted bivariate tests by location. Categorical variables were assessed using chi-squared tests, while continuous variables were analyzed using t-tests. Following this, location-based disparities in the perceived impact of COVID-19 on mental health were explored. Lastly, the risk associated with a major negative perceived impact on mental health due to lockdown and curfew measures was estimated using ordered logistic regressions First, to ensure reliability, collinearity was examined through crosstabs. Next, stratified models were run based on respondent location (rural, small/medium, urban) to evaluate potential variations in predictors influencing the perceived impact on mental health.

## Results

After excluding respondents with missing variables, 23,671 women were included in the study (6,131 (25.9%) in rural areas, 9,147 (38.6%) in small or medium-sized towns and 8,393 (35.5%) in urban areas). As shown in Table 1, the mean age was 45.7 years in both rural and urban areas, reaching an upper limit of 91 years.

**Table 1:** Sample characteristics by location

	Rural	Small/Medium	Urban	Rural vs Small/Medium	Rural vs
					Urban
				p-value	p-value
Age *Mean* *(Standard deviation)*	45.7 (16.6)	46.2 (16.6)	45.7 (16.6)	*	
Disabled	7.0%	6.1%	5.6%	*	***
Employment status					
Self-employed	8.2%	8.6%	9.4%		**
Employee	48.9%	49.8%	54.5%		***
Manual worker	4.7%	5.7%	3.9%	**	*
Not working	38.3%	35.9%	32.2%	***	***
Educational attainment					
Never in full-time education	2.0%	2.3%	1.6%		
Primary education	4.1%	3.3%	2.0%	**	***
Secondary education	41.4%	36.1%	28.2%	***	***
Tertiary education	52.6%	58.3%	68.1%	***	***
Household type					
Couple with children	37.9%	33.3%	30.3%	***	***
Couple without children	23.6%	23.9%	24.0%		
Single parent with children	7.2%	8.9%	8.5%	***	***
Single without children	10.7%	14.4%	18.5%	***	***
Multi-generational household	13.4%	10.8%	9.4%	***	***
Co-living	1.4%	2.0%	3.4%	**	***
Other	5.8%	6.8%	5.9%	*	
Violence	74.7%	74.2%	75.4%		
*N* = 23,671	6,131	9,147	8,393		

Results of t tests and chi-squared tests are significant at: *p < 0.05, **p < 0.001, ***p < 0.001

In rural areas, a higher percentage of women reported disabilities (7%) compared to those in small or medium-sized towns (6.1%, *p* < 0.05) and urban areas (5.6%, *p* < 0.001). Similarly, a larger proportion of non-working women was found in rural areas (38.3%) compared to small or medium-sized towns (35.9%, *p* < 0.001) and urban areas (32.2%, *p* < 0.001).

Although the prevalence of tertiary education completion was notable in rural areas at 52.6%, it was comparatively lower than in small or medium-sized towns (58.3%, *p* < 0.001) and urban areas (68.1%, *p* < 0.001). Moreover, a higher percentage of women in rural areas were in couples with children (37.9%) compared to small or medium-sized towns (33.3%, *p* < 0.001) and urban areas (30.3%, *p* < 0.001). Conversely, urban areas had a greater percentage of single women without children (18.5%) compared to small or medium-sized towns (14.4%, *p* < 0.001) and rural areas (10.7%, *p* < 0.001).

In terms of perceptions of violence against women due to the pandemic, around three-quarters of respondents across all locations perceived an increase, with no significant differences based on their residential location.

Across rural, small/medium-sized towns, and urban areas, differences in the perceived negative impact on mental health were statistically significant (Table 2). Women living in urban areas generally reported a higher perceived negative impact on mental health (Mode = 4, Median = 3) compared to women in rural areas (Mode = 3, Median = 3) and to women in small/medium-sized towns (Mode = 3, Median = 3). Following the application of the Kruskal–Wallis H test, we found a significant difference in the perceived negative impact on mental health across areas (*p* < 0.001). After conducting the Kruskal–Wallis H test, we proceeded with Dunn´s post-hoc test to examine pairwise differences between groups, using the Bonferroni correction for adjusting the p-values. The adjusted p-values indicated significant differences between rural and urban areas(*p* < 0.001), between rural areas and small/medium-sized towns (*p* < 0.01) and between small/medium-sized towns and urban areas (*p* < 0.05).

**Table 2:** Perceived impact of lockdown and curfew measures on women's mental health by location

	Rural	Small/Medium	Urban
Mean	3.02	3.09	3.13
SD	1.43	1.40	1.40
Mode	3	3	4
Median	3	3	3
Kruskal–Wallis H test	21.94***		
Dunn´s test: Rural vs. Small/Medium	-2.80**		
Dunn´s test: Rural vs. Urban	-4.68***		
Dunn´s test: Small/Medium vs. Urban	-2.14*		

Results of tests are significant at: *p < 0.05, **p < 0.001, ***p < 0.001

**Fig. 1:**
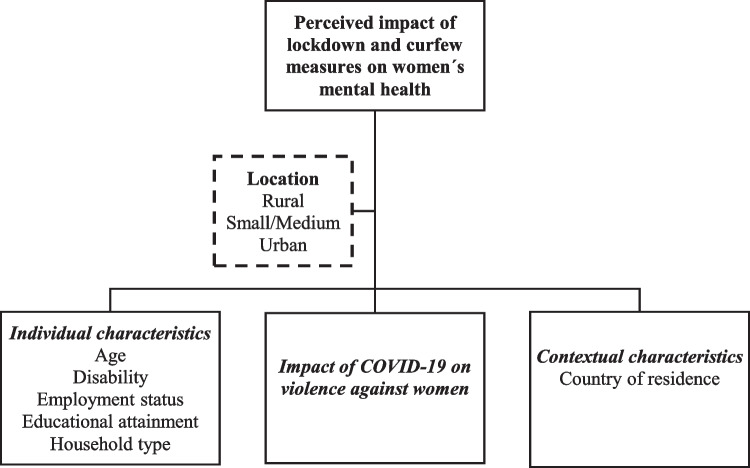
Conceptual model of factors affecting the perceived impact of lockdown and curfew measures on women´s mental health. Based on Peterson et al. (2009)

Figure 1 summarizes our theoretical model, and Table 3 presents the results of the ordered logistic regression model predicting the perceived impact on women's mental health due to lockdown and curfew measures, stratified by rural, small/medium-sized, and urban locations. It is important to note that odds ratios cannot be interpreted as total-effects (Westreich and Greenland 2013).

**Table 3:** Regression analysis predicting the perceived impact of lockdown and curfews on women´s mental health by location

	Rural		Small/Medium		Urban	
	aOR	95% CI	aOR	95% CI	aOR	95% CI
Age	0.97***	0.97–0.98	0.98***	0.97–0.98	0.97***	0.97–0.98
Disabled	1.44***	1.20–1.73	1.15	0.98–1.35	1.36***	1.15–1.62
Employment status: Ref. Self-employed						
Employee	1.08	0.91–1.28	1.02	0.89–1.17	0.89	0.78–1.02
Manual worker	1.29	0.98–1.69	1.17	0.95–1.44	0.93	0.73–1.18
Not working	1.06	0.89–1.27	0.93	0.81–1.08	0.90	0.78–1.05
Educational attainment: Ref. Never in full-time education						
Primary education	1.26	0.85–1.87	0.94	0.68–1.29	0.90	0.60–1.36
Secondary education	1.08	0.78–1.50	0.89	0.69–1.14	0.97	0.70–1.33
Tertiary education	1.02	0.73–1.42	0.97	0.76–1.24	0.97	0.71–1.33
Household type: Ref. Couple with children						
Couple without children	0.97	0.85–1.10	0.97	0.88–1.07	0.89*	0.80–0.99
Single parent with children	1.08	0.90–1.29	1.06	0.92–1.21	1.03	0.88–1.19
Single without children	1.01	0.86–1.19	1.04	0.92–1.17	0.92	0.82–1.04
Multi-generational household	1.06	0.92–1.23	1.00	0.88–1.14	1.02	0.88–1.18
Co-living	1.21	0.83–1.75	1.08	0.83–1.41	1.07	0.86–1.34
Other	1.10	0.89–1.36	0.95	0.81–1.12	0.97	0.81–1.16
Violence	1.56***	1.40–1.74	1.29***	1.18–1.41	1.29***	1.17–1.41
Country dummies Ref. France						
Austria	0.93	0.72–1.21	0.76	0.58–1.01	0.74	0.53–1.02
Belgium	0.84	0.66–1.08	0.69***	0.54–0.88	0.78	0.55–1.10
Bulgaria	0.92	0.56–1.51	0.78	0.60–1.01	0.83	0.61–1.12
Cyprus	1.14	0.77–1.67	1.14	0.84–1.57	1.17	0.81–1.68
Czech Republic	1.12	0.85–1.48	0.91	0.72–1.16	0.90	0.65–1.26
Germany	0.75*	0.57–0.97	0.65***	0.51–0.84	0.72*	0.52–1.00
Denmark	0.46***	0.34–0.62	0.47***	0.37–0.61	0.52***	0.38–0.71
Estonia	0.46***	0.34–0.63	0.42***	0.31–0.56	0.38***	0.28–0.52
Spain	1.09	0.79–1.50	1.17	0.93–1.48	1.20	0.88–1.63
Finland	0.62**	0.44–0.87	0.49***	0.38–0.64	0.56***	0.41–0.76
Greece	1.64**	1.11–2.42	1.27	0.97–1.67	1.55***	1.16–2.08
Croatia	1.09	0.80–1.49	0.81	0.63–1.04	0.82	0.59–1.13
Hungary	1.22	0.89–1.67	0.78	0.61–1.01	0.80	0.58–1.11
Ireland	0.66***	0.51–0.85	0.73**	0.56–0.94	0.72*	0.52–0.99
Italy	1.25	0.92–1.70	1.17	0.94–1.47	1.13	0.82–1.56
Lithuania	0.66*	0.46–0.95	0.47***	0.36–0.61	0.50***	0.37–0.69
Luxembourg	0.73	0.52–1.03	0.80	0.59–1.09	0.79	0.52–1.22
Latvia	0.65**	0.47–0.90	0.45***	0.34–0.60	0.64***	0.47–0.86
Malta	0.73	0.52–1.03	0.59***	0.45–0.78	0.49***	0.32–0.75
The Netherlands	0.69**	0.52–0.91	0.58***	0.45–0.75	0.59***	0.41–0.85
Poland	1.80***	1.31–2.48	1.35*	1.05–1.75	1.38*	1.00–1.89
Portugal	1.24	0.91–1.68	0.86	0.68–1.10	0.93	0.67–1.28
Romania	0.77	0.52–1.13	0.56***	0.44–0.72	0.63***	0.46–0.85
Sweden	0.58***	0.43–0.78	0.52***	0.41–0.68	0.55***	0.40–0.75
Slovenia	1.07	0.83–1.39	1.03	0.79–1.34	1.38	0.93–2.03
Slovakia	0.90	0.69–1.16	0.94	0.73–1.21	0.72	0.49–1.04
*N*	6,131		9,147		8,393	
Log-likelihood	-9,484.69		-14,166.46		-12,952.29	
*p*-value	< 0.001		< 0.001		< 0.001	

Results are generated from ordered logistic regression models predicting the perceived impact on mental health (1 = minor negative impact; 5 = major negative impact). Results are significant at: * *p* < 0.05, ** *p* < 0.01, *** *p* < 0.001

Regarding individual factors, both age and disability showed associations with perceiving a negative impact on mental health. Age displayed consistent odds across rural areas (aOR 0.97, 95% CI = 0.97–0.98), small or medium-sized towns (aOR 0.98, 95% CI = 0.97–0.98), and urban areas (aOR 0.97, 95% CI = 0.97–0.98). Concerning disability, the odds were higher in rural areas (aOR 1.44, 95% CI = 1.20–1.73) compared to urban areas (aOR 1.36, 95% CI = 1.15–1.62). Within the urban cohort, women in a couple without children were less likely to perceive a negative impact on mental health (aOR 0.89, 95% CI = 0.80–0.99) compared to those in a couple with children.

Respondents perceiving an increase in violence against women due to COVID-19 were more inclined to report a negative mental health impact. The odds ratio was higher for rural areas (aOR 1.56, 95% CI = 1.40–1.74) than for urban areas (aOR 1.29, 95% CI = 1.17–1.41).

Moreover, significant differences were observed across countries. In some countries, such as Estonia (aOR 0.38, 95% CI = 0.28–0.52), Malta (aOR 0.49, 95% CI = 0.32–0.75), Lithuania (aOR 0.50, 95% CI = 0.37–0.69), Denmark (aOR 0.52, 95% CI = 0.38–0.71), Sweden (aOR 0.55, 95% CI = 0.40–0.75), Finland (aOR 0.56, 95% CI = 0.41–0.76), the Netherlands (aOR 0.59, 95% CI = 0.41–0.85), Latvia (aOR 0.64, 95% CI = 0.47–0.86), and Romania (aOR 0.63, 95% CI = 0.46–0.85), women in urban areas were less prone to perceive a negative mental health impact. Conversely, urban women in Greece were more likely to perceive such an impact (aOR 1.55, 95% CI = 1.16–2.08).

## Discussion

Numerous studies have detailed the pandemic's adverse effects on women's mental well-being. This cross-sectional study aimed to explore how women's perception of the impact of lockdowns and curfews on mental health varied based on their residing location and other contextual and individual factors. Women living in urban areas generally reported a higher perceived negative impact on mental health compared to women in rural areas or in small/medium-sized towns, consistent with prior research linking urban residency to poorer mental health outcomes (Greteman et al. 2022; Liu et al. 2021; Pérès et al. 2021).

We examined three primary factors affecting mental health: individual characteristics, contextual elements, and opinions regarding COVID-19's impact on violence against women. Disabled women were more likely to perceive a negative mental health impact, possibly due to encountering greater challenges coping with the unprecedented situation, especially pronounced in rural areas compared to urban setting (Peters 2020; Sage et al. 2019; Zhao et al. 2019). Interestingly, older age appeared to play a "protective" role, reducing the likelihood of perceiving a negative impact on mental health. This might relate to younger women's perceived loss of social interaction or more uncertain work conditions during the pandemic (Etheridge and Spantig 2022; Pieh et al. 2020).

In contrast to previous studies that associated lower educational levels with pandemic-related impacts (Albrecht 2022), our findings did not reveal any clear relationship between educational attainment and the effects of lockdowns and curfews on mental health. Although most studies suggest that employment promotes women´s mental health, this benefit is contingent upon the presence or absence of children and upon the type of job (Bruns and Pilkauskas 2019; Jacobs et al. 2016; Lefkowitz and Armin 2021). Studies conducted in different countries have confirmed that caring for children during the pandemic had a negative impact on mental health (Almeida et al. 2020; Ben Brik et al. 2022; Cheng et al. 2021; Russell et al. 2022; Sevilla and Smith 2020). In many households, lockdown and curfews meant more childcare for women (Borrescio-Higa and Valenzuela 2021; Sevilla and Smith 2020) and working parents who had children experienced a more pronounced degree of financial distress compared to working parents without children (Cheng et al. 2021). In our analysis we found that, in urban areas, women in childless couples were less likely to perceive a negative impact on mental health compared to women in couples with children.

The perceived increase in violence against women during the pandemic influenced the impact of lockdowns and curfews on mental health, especially in rural areas, consistent with previous research highlighting a surge in such violence (Ben Brik et al. 2022; de Baumont et al. 2023; Sediri et al. 2020; Shewangzaw Engda et al. 2022). Additionally, differences across countries underscored the role of contextual factors in explaining variations in the impact of lockdowns and curfews on mental health.

However, our study has limitations worth mentioning. Using a single self-reported question to evaluate the impact on mental health may introduce biases, including cognitive or memory biases. A validated psychometric instrument would have been more adequate to study the effects on mental health. The cross-sectional nature of our data prevents establishing causality, and omitted variable bias might exist despite adjusting for relevant covariates. Additionally, the use of online panels may have selected participants biased towards better health. Future studies should consider more comprehensive adjustments and validated instruments to further explore these associations.

## Conclusions

Our study highlights variations in the perceived impact of lockdown and curfew measures on women´s mental health across urban and rural areas. To unravel potential reasons, we delved into individual factors like age, disability, employment, education, household dynamics, alongside considering perspectives on COVID-19's impact on violence against women and country-specific contexts. Our findings unveil that the mental health repercussions of lockdowns and curfews were shaped not just by individual characteristics (such as age, disability, or household structure) but also influenced by the pandemic's effect on violence against women and contextual aspects tied to country-specific settings. Hence, future studies examining the impact of COVID-19 on mental health need to account for variations across different settings.

## Data Availability

The data used in this study are publicly available at https://europa.eu/eurobarometer/surveys/detail/2712.
